# Comparison of Controlling Nutritional Status Score with Bedside Index for Severity in Acute Pancreatitis Score and Atlanta Classification for Mortality in Patients with Acute Pancreatitis

**DOI:** 10.3390/jcm13123416

**Published:** 2024-06-11

**Authors:** Betül Çavuşoğlu Türker, Süleyman Ahbab, Fatih Türker, Emre Hoca, Ece Çiftçi Öztürk, Atay Can Kula, Hüseyin Öztürk, Ayşe Öznur Urvasızoğlu, Nilsu Kalaycı, Erdem Koçak, Merve Bulut, Özge Yasun, Hayriye Esra Ataoğlu

**Affiliations:** 1 Department of Internal Medicine, Haseki Health Training and Research Hospital, University of Health Sciences Türkiye, Istanbul 34130, Türkiye; cavusoglubetul@hotmail.com (B.Ç.T.); drsahbab@gmail.com (S.A.); emre.hoca@hotmail.com (E.H.); eciftci3506@gmail.com (E.Ç.Ö.); oznurvasiz@gmail.com (A.Ö.U.); dr.nilsukalayci@gmail.com (N.K.); eataoglu@gmail.com (H.E.A.); 2 Department of Internal Medicine, Medical Faculty, Balıkesir University, Balıkesir 10050, Türkiye; ataycankula@gmail.com; 3 Department of Internal Medicine, Başakşehir Çam & Sakura City Hospital, University of Health Sciences Türkiye, Istanbul 34480, Türkiye; huseyinozturkdr@gmail.com; 4 Department of Internal Medicine, Liv Hospital, Istınye University, Istanbul 34010, Türkiye; kocak67@hotmail.com; 5 Department of Internal Medicine, Gaziosmanpaşa Taksim Health Training & Research Hospital, University of Health Sciences Türkiye, Istanbul 34480, Türkiye; merve3792@hotmail.com; 6 Internal Medicine Department, Hakkari State Hospital, Hakkari 30000, Türkiye; ozgeyasun@gmail.com

**Keywords:** CONUT score, prognostic value, mortality, acute pancreatitis

## Abstract

**Background/Objectives**: Acute pancreatitis (AP) is characterized by pancreatic gland inflammation, and its clinical course ranges from mild to severe. Predicting the severity of AP early and reliably is important. In this study, we investigate the potential use of the Controlling Nutritional Status (CONUT) score as a prognostic marker in acute pancreatitis. **Methods**: We examined 336 patients who had been hospitalized with an AP diagnosis in the internal medicine clinic. The patients included in the study were followed up for 5 years. The study analyzed the specific variables of age, gender, and AP etiology as recorded biochemical parameters for all study participants and calculated the effects of age, sex, Bedside Index of Severity in AP (BISAP), the revised Atlanta classification, and the CONUT score on mortality. **Results**: When compared with surviving patients, non-surviving patients had higher scores for BISAP, CONUT, and the Atlanta Classification (*p ˂* 0.001). In the non-surviving group, hemoglobin, lymphocyte, and albumin levels were significantly lower and creatinine, uric acid, and procalcitonin levels were significantly higher compared to the surviving group (*p* ˂ 0.001, 0.003, ˂0.001, ˂0.001, 0.005, ˂0.001, respectively). The multivariate analysis showed a significant association of mortality with age, CONUT, and BISAP scores (*p* ˂ 0.003, 0.001, 0.012 respectively). The CONUT score was separated into two groups based on the median value. The predicted survival time in the group with a CONUT score > 2 (53.8 months) was significantly lower than in the group with a CONUT score ≤ 2 (63.8 months). The cumulative incidence of all-cause mortality was significantly higher in the patients with higher CONUT scores. **Conclusions**: This study has assigned the CONUT score as an independent risk factor for mortality in AP.

## 1. Introduction

Acute pancreatitis (AP) is characterized by pancreatic gland inflammation. Its clinical course ranges from mild to severe, and its incidence is gradually increasing. AP is a condition characterized by elevated pancreatic enzymes in the blood and urine as laboratory findings and as abdominal pain on physical examination. The etiology of AP is multifactorial, with gallstones and alcohol being the most common causes. Its etiology also includes hypertriglyceridemia, hypercalcemia, infection, trauma, and autoimmune diseases [1,2]. Acute pancreatitis may progress with recurrent attacks. This condition can cause permanent damage to the pancreas, resulting in chronic pancreatitis or pancreatic insufficiency [3,4]. Although no specific treatment exists for acute pancreatitis, its course can be fatal or morbid.

Predicting the severity of AP early and reliably is important [5]. Many multifactorial scoring systems have been developed since the 1979s to predict the severity and prognosis of AP. The Bedside Index for Severity in Acute Pancreatitis (BISAP) scoring system is applied within the first 24 h after admission and evaluates five parameters (blood urea nitrogen [BUN], mental status impairment, systemic inflammatory response syndrome [SIRS], age, and pleural effusion), each worth one point [6]. The most important feature of the BISAP scoring system is its early and easy applicability. According to the revised Atlanta Classification, patients with AP are considered to have mild AP if no organ failure and no local or systemic complications have occurred. Those with transient organ failure and/or local or systemic complications that resolve within 48 h are considered to have moderate AP, while those with ongoing organ failure that does not resolve within 48 h are considered to have severe AP [7].

Nutritional support for patients with acute pancreatitis varies in accordance with the disease severity. Mild and moderate pancreatitis has little impact on nutritional status and metabolism. These patients can generally return to their normal nutrition within 3–7 days. In severe AP, a negative nitrogen balance may occur due to a protein energy deficit and increased protein catabolism [8,9]. This may adversely affect the patient’s nutritional status and disease progression. Studies have shown that patients with a negative nitrogen balance have mortality rates 10 times higher than those with a normal nitrogen balance [10,11].

Recently, inflammatory and nutritional status markers measured using parameters, such as the Controlled Nutritional Status (CONUT) score, have been shown to be successful in predicting poor prognosis in various diseases [12,13]. These inflammatory and nutritional status parameters are based on serum and/or peripheral blood counts and are easily measured in daily clinical practice [14,15].

In this study, we investigate the potential use of the CONUT score as a prognostic marker in AP.

## 2. Materials and Methods

### 2.1. Patients

A total of 456 patients diagnosed with acute pancreatitis (AP) were included. The inclusion criteria required patients to meet at least two of the three diagnostic criteria for AP: abdominal pain, serum lipase or amylase levels at least three times the upper normal limit, and characteristic imaging findings. Exclusion criteria included patients under 18 (number of cases = 2), pregnant women (number of cases = 1), patients with delayed hospital admission (>24 h) (number of cases = 10), those with solid or hematological malignancies (number of cases = 10), readmissions (number of cases = 69), cases with insufficient data (number of cases = 12), and those diagnosed with chronic pancreatitis (number of cases = 16). The patients were followed for an average of 34 months (range: 0–66 months). Patients for whom primary endpoints and all-cause mortalities apart from unnatural deaths (e.g., accident, suicide, murder) fell within the five-year follow-up period were also excluded. The patients’ post-discharge mortality data were obtained. Patients’ demographic information, past medical history, mortality data, and laboratory examination were taken for the study from the electronic hospital management system dispensing records and databases of the Haseki Training and Research Hospital.

### 2.2. Ethical Aspects

This study was conducted with the approval of the local Ethics Committee of the Haseki Training and Research Hospital, University of Health Sciences, Istanbul, Turkey (Approval No. 66-2023-Approval Date 23 March 2023), in accordance with the principles of the Declaration of Helsinki and good clinical practice guidelines.. Informed consent was obtained from all patients during hospitalization. Data on patient demographics, medical history, laboratory results, and follow-up information were collected from the hospital’s electronic management system and the Turkish National Mortality Registry.

### 2.3. Study Outcome

The primary outcome was to evaluate the prognostic value of the Controlling Nutritional Status (CONUT) score in predicting mortality in patients with AP. Secondary outcomes included comparing the CONUT score with the Bedside Index for Severity in Acute Pancreatitis (BISAP) score and the Revised Atlanta Classification.

### 2.4. Study Design

This retrospective cohort study analyzed patients hospitalized with an AP diagnosis from 1 January 2018 to 1 January 2023. All patients were assessed using CONUT, BISAP, and Revised Atlanta Classification scores. Laboratory parameters, including hemoglobin, white blood cell count, neutrophils, lymphocytes, creatinine, uric acid, glucose, AST, ALT, triglycerides, cholesterol, albumin, and procalcitonin levels, as well as age, gender, and AP etiology, were recorded. Blood samples were taken after a 12 h fasting period on the first day of hospitalization. All patients performed a zero diet after hospitalization and returned to their normal nutrition in 3–7 days. BISAP scores were calculated for all participants, and each criterion was worth one point: BUN > 25 mg/dL (8.92 mmol/L), impaired mental status (defined as disorientation, lethargy, somnolence, coma, or stupor), ≥2 SIRS criterion, age > 60 years, pleural effusion present). The patients were evaluated according to the Revised Atlanta Classification for AP, after which the severity of AP was determined [7,16]. Mild acute pancreatitis is characterized by the absence of organ failure and the presence of few or no local or systemic complications. Moderately severe acute pancreatitis is characterized by transient organ failure, which typically resolves within 48 h, and/or local or systemic complications without persistent organ failure (>48 h). Severe acute pancreatitis is characterized by persistent organ failure that may involve one or multiple organs. Local complications of acute pancreatitis include acute peripancreatic fluid collection, pancreatic pseudocyst, acute necrotic collection, and walled-off necrosis. Organ failure is defined as a score of two or more for any one of three organ systems (respiratory, cardiovascular, or renal) using the modified Marshall scoring system. Systemic complications were identified using the definition of exacerbation of existing chronic diseases due to AP as defined in the Revised Atlanta Classification. CONUT scores were formulated for all participants as the sum of the scores based on the serum albumin (0, 2, 4, 6), total cholesterol concentration, and total lymphocyte count (0, 1, 2, or 3 for each) and classified as normal, mild, moderate, or severe for the respective scores of 0–1, 2–4, 5–8, and 9–12. The effects of age, sex, BISAP score, Atlanta Classification, CONUT score, and biochemical parameters were compared over the surviving and non–surviving groups.

### 2.5. Follow-Up

Patients were followed for a mean of 34 months, with the minimum follow-up being 0 months and the maximum being 66 months. Post-discharge mortality data were obtained from the hospital data processing system and verified through the Turkish National Mortality Registry.

### 2.6. Radiological Evaluation

Abdominal CT scans were performed using a 64-detector Philips CT device (Brilliance, Philips Medical Systems, Cleveland, OH, USA). Abdominal CT scans were conducted according to the routine intravenous contrast-enhanced protocol, which includes upper abdominal CT without axial contrast and whole abdominal CT taken in the portal venous phase at 60 s. Abdominal ultrasonography was performed as the first assessment for all study patients with acute pancreatitis, followed by abdominal CT. The abdominal CT was performed 6–72 h after the patients were admitted to the hospital. Abdominal CT images were reviewed retrospectively and obtained from pre-contrast axial sections using the INFINITT PACS version 3.0.11.4 (INFINITT Healthcare Co. Ltd., Seoul, Republic of Korea) by two expert radiologists with five years of experience. The radiologists were blinded to the patients’ clinical data. Attenuation measurements of the pancreas at the caput, corpus, and cauda were recorded.

### 2.7. Statistical Analysis

Means, standard deviations, medians, minima, and maxima values, frequencies, and percentages were used for the descriptive statistics. The distributions of the variables were checked using the Kolmogorov–Smirnov test. The independent samples *t*-test and Mann–Whitney U test were used to compare the quantitative data. The chi-squared test was used to compare the qualitative data. Receiver operating characteristic (ROC) analysis was used to show the effect level. The effect level was also investigated using univariate and multivariate logistic regressions. The Kaplan–Meier estimator was used regarding the survival analysis. SPSS 28.0 was used for the statistical analyses.

## 3. Results

We retrospectively analyzed a total of 336 patients. Table 1 shows the patients’ baseline characteristics. The age, CONUT score, Atlanta Classification, and BISAP score were significantly higher in the non-surviving group than in the surviving group (*p ˂* 0.001). In the non-surviving group, hemoglobin, lymphocyte, and albumin levels were significantly lower and creatinine, uric acid, and procalcitonin levels were significantly higher compared to the surviving group (Table 1). With regard to the AP etiologies, 127 were biliary, 19 were alcoholism, 28 were hypertriglyceridemia, 8 were autoimmune, 4 were viral infections, and 149 were other etiological factors (e.g., post ERCP, drugs; see Table 2).

The univariate analysis associated mortality with the age, CONUT score, Atlanta Classification, BISAP score, and hemoglobin, lymphocyte, creatinine, and albumin levels. The multivariate analysis showed a significant association of mortality with the CONUT score, BISAP score, and age (Table 3), with a higher CONUT score, BISAP score, and age showing a very close relationship in terms of mortality risk in AP patients (age: M = 1.045, 95% CI [1.015, 1.076], *p* = 0.003; CONUT score: M = 1.344, 95% CI [1.131, 1.598], *p* = 0.001; BISAP score: M = 1.622, 95% CI [1.114, 2.363], *p* = 0.012).

**Figure 1:**
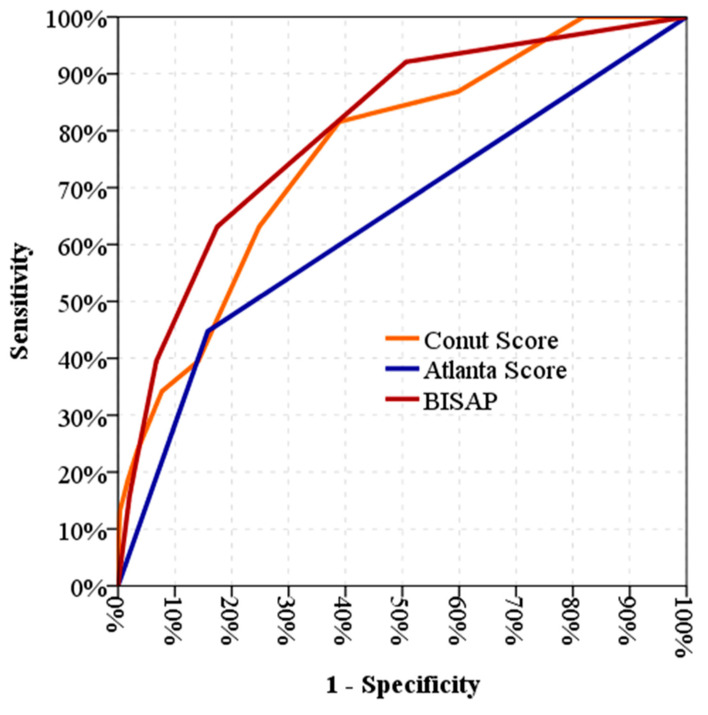
ROC curve for CONUT scores, Atlanta classifications, and BISAP scores based on mortality. Negative predictive value (NPV) and positive predictive value (PPV) for varying prevalence values. The NPV and PPV were calculated, based on the observed sensitivity and specificity in the blinded validation set, for varying prevalence values. Red line: the entire validation set for BISAP scores (sensitivity: 92.1%, specificity: 84.2%), calculated NPV: 98.8%; calculated PPV: 42.7%. Orange line: the entire validation set for CONUT scores (sensitivity: 81.6%, specificity: 61.1%), calculated NPV: 96.3%; calculated PPV: 21.1%. Blue line: the entire validation set for Atlanta scores (sensitivity: 44.7%, specificity: 50.3%), calculated NPV: 87.7%; calculated PPV: 10.3%.

A ROC curve was drawn among the CONUT score, BISAP score, and Atlanta Classification as shown in Figure 1. The area under the curve (AUC) for the CONUT score was 0.762 (95% CI [0.685, 0.840]). The AUC for the BISAP score was 0.801 (95% CI [0.728, 0.875]). The AUC for the Atlanta Classification was 0.645 (95% CI [0.543, 0.747]).

**Figure 2:**
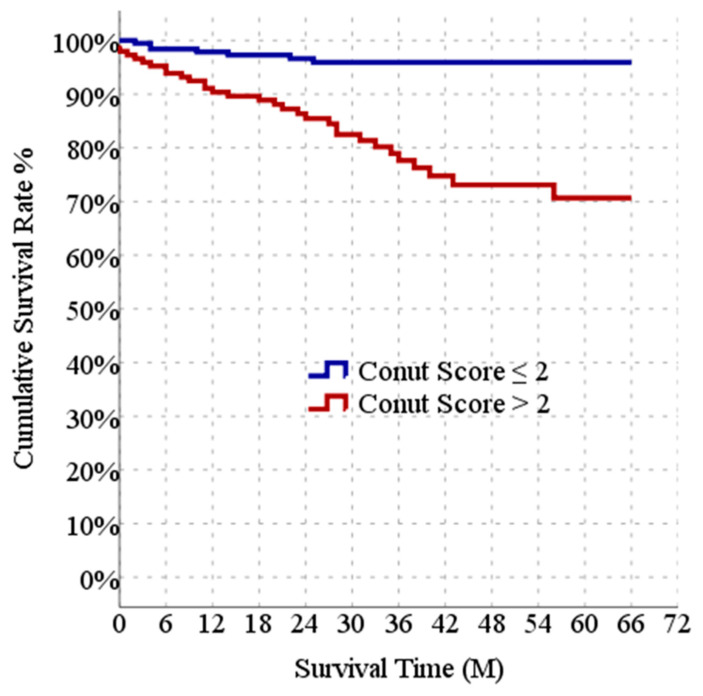
Kaplan–Meier survival plots compared using the log-rank test (*p* < 0.0001).

The CONUT scores were divided into two groups according to the median value. Kaplan–Meier curves illustrate the incidence of all-cause death categorized based on CONUT ≤ 2 and CONUT > 2 in AP patients. The predicted survival time in the group with CONUT scores > 2 (53.8 months) was significantly lower (*p* < 0.05) than in the group with CONUT scores ≤ 2 (63.8 months). The cumulative incidence of all-cause mortality was significantly higher in the patients with higher CONUT scores (Figure 2).

## 4. Discussion

In this study, we have investigated the utility and predictive power of the CONUT score for evaluating mortality in AP patients. We showed CONUT scores to be significantly higher in patients from the non-surviving group. Moreover, a higher CONUT score was an independent risk factor for mortality in AP patients.

Few studies have been conducted on the relationship between the CONUT score and mortality in AP patients. Lvyuan Shi et al. found the CONUT score to be an independent predictor of short-term prognosis in patients with severe AP [17,18]. In our current study, the CONUT score was related to mortality in AP patients. Liu C et al. assigned the CONUT score as a predictive marker of in-hospital mortality in older patients [19]. Unlike other studies, we found a CONUT score greater than 2 to be an independent predictor of mortality in young patients.

The Atlanta Classification and BISAP score have long been used safely to determine the prognosis of AP. The BISAP score and Atlanta Classification are based on a variety of patient clinical findings, while the CONUT score is based on serum albumin levels, the total cholesterol (TC) concentration, and total lymphocyte count (TLC) [20]. CONUT is a nutrition-related tool that is easy, low cost, and simple to evaluate; it is also calculated using objective parameters. Recent studies have determined the three components of the CONUT score that are associated with prognosis and mortality [20,21].

Low serum albumin levels are known to be associated with increased mortality in hospitalized patients. As a component of the CONUT score, serum albumin levels are an important determinant of mortality and in-hospital complications in hospitalized patients [22]. Two main mechanisms are said to be associated with serum albumin levels and increased mortality. First, albumin exhibits structurally specific antioxidant properties, and hypoalbuminemia can accordingly cause cellular oxidative damage and apoptosis [23]. Second, serum albumin levels provide information about the status of systemic protein metabolism and inflammation [24]. Although whether or not serum albumin levels directly specify malnutrition is unclear, studies have shown low serum albumin levels to serve as a marker of inflammation associated with the nutritional status and risk of developing mortality [25]. In our study, low serum albumin levels were related to high mortality, which is consistent with the literature.

Total cholesterol (TC) is one of the constituent parameters of the CONUT score. Studies have shown low TC to be associated with a higher risk of death in patients. Low TC levels represent a poor nutritional status and increased inflammation in inpatients [26,27].

The last parameter of the CONUT score is the total lymphocyte count (TLC). Low TLC levels are also known to be associated with a reduced immune status and inflammatory state and are also known to be associated with higher mortality in inpatients and to be a prognostic indicator [28]. A similar study concluded a TLC less than 0.8 G/L to be associated with an increased risk of death, readmission, and length of stay in hospitalized patients [29]. This study similarly found that CONUT score, as a combination of these three parameters, to be a good marker for predicting mortality.

Recent studies have determined the CONUT score to have a predictive effect on mortality in various diseases This study found the CONUT score to be effective in determining mortality in AP patients. Additionally, our study found the CONUT score to be as effective as BISAP scores and the Atlanta Classification in predicting mortality.

This study has several strengths. To our knowledge, it is the first to investigate the short- and long-term prognostic value of the CONUT score in hospitalized patients with a diagnosis of AP in comparison to the BISAP score and Atlanta Classification. The significance of the CONUT score at admission is an important finding that helps clinicians identify patients who are at an increased risk of mortality and who may benefit from interventions, such as early nutritional support.

Research on this subject is quite new and limited. When evaluating the literature, studies were observed to involve chronic diseases and malignancies. The feature that distinguishes our study from others is the high number of cases and long follow-up period.

Our study has some limitations. First, our study is retrospective and it was performed in a single center. Secondly, a limited number of hematological and biochemical markers have been studied, which may limit the implications of the findings.

## 5. Conclusions

Our study indicates the clinical importance of the CONUT score as a novel marker for predicting mortality in AP patients. We found the CONUT score to be an independent risk factor for mortality, similar to the BISAP score and Atlanta Classification. The CONUT score has been identified as a novel prognostic marker for all-cause mortality in AP and may serve as a helpful, inexpensive, and noninvasive prognostic mortality marker for AP patients. Further studies are needed to confirm our findings.

## Figures and Tables

**Table 1:** Patients’ baseline characteristics.

	Survival	Non-Survival	*p* Value
Gender Females, *n* (%)	150 (50.3%)	14 (36.8%)	0.117
Males, *n* (%)	148 (49.7%)	24 (63.2%)	
Age (year)	51.5 (39–63.3) ^a^	73 (23–91) ^a^	**˂0.001**
CONUT Score	2 (1.0–3.3) ^a^	4 (3.0–6.3) ^a^	**˂0.001**
Atlanta Classification: Mild	251 (84.2%)	21 (55.3%)	**˂0.001**
Moderate-Severe	47 (15.8%)	17 (44.7%)	
BISAP 0	147 (49.3%)	3 (7.9%)	**˂0.001**
1	99 (33.2%)	11 (28.9%)	
2	32 (10.7%)	9 (23.7%)	
3	14 (4.7%)	9 (23.7%)	
4	6 (2.0%)	6 (15.8%)	
Hemoglobin (g/dL)	12.8 (11.6–14.0) ^a^	11.7 (9.9–12.7) ^a^	**0.001**
White blood cell (×10^9^/L)	9.4 (7–12) ^a^	10.6 (7.2–14.8) ^a^	0.211
Neutrophil (×10^9^/L)	6.8 (4.3–9.4) ^a^	8 (4.5–12.7) ^a^	0.116
Lymphocyte (×10^9^/L)	1.6 (1.19–2.09) ^a^	1.3 (0.76–1.78) ^a^	**0.003**
Creatinine (mg/dL)	0.7 (0.58–0.82) ^a^	1 (0.7–1.75) ^a^	**˂0.001**
Uric Acid (mg/dL)	4.4 (3.6–5.5) ^a^	5.7 (4.1–6.6) ^a^	**0.005**
Glucose (mg/dL)	106 (89–145) ^a^	127.5 (91.3–173) ^a^	0.083
AST (U/L)	26.5 (17–81.5) ^a^	37.5 (24.8–93.3) ^a^	0.170
ALT (U/L)	30.5 (15–121.3) ^a^	33 (19.5–80.5) ^a^	0.950
Triglyceride (mg/dL)	112 (75–179.5) ^a^	102 (74–150) ^a^	0.407
Cholesterol (mg/dL)	167 (135–201) ^a^	175 (134.5–206) ^a^	0.869
Albumin (g/dL)	37 (33–39) ^a^	31 (12.5–35.5) ^a^	**˂0.001**
Procalcitonin (µg/L)	0.1 (0.1–0.6) ^a^	0.6 (0.3–1.6) ^a^	**˂0.001**

Note: Statistical significance is shown in bold-faced type (*p* < 0.05). ^a^: Median (25th–75th percentile). Abbreviations: CONUT= Controlling Nutritional Status; BISAP = Bedside Index for Severity in Acute Pancreatitis; AST = aspartate transferase; ALT = alanine aminotransferase.

**Table 2:** Etiology of acute pancreatitis.

Disease	Number
Alcoholism	19 (5.6%)
Gallstone	127 (37.7%)
Hypertriglyceridemia	28 (8.3%)
Hypercalcemia	1 (0.2%)
Autoimmune	8 (2.4%)
Viral Infections	4 (1.2%)
Other (post ERCP, drugs, etc.)	149 (44.8%)

Abbreviations: ERCP = Endoscopic retrograde cholangiopancreatography.

**Table 3:** Univariate and multivariate analysis of the variables associated with mortality.

	Univariate Model					Multivariate Model				
	*M*	95% CI			*p*	*M*	95% CI			*p*
Age	1.077	1.050	−	1.105	**0.000**	1.045	1.015	−	1.076	**0.003**
CONUT Score	1.541	1.324	−	1.794	**0.000**	1.344	1.131	−	1.598	**0.001**
Atlanta Score	4.323	2.123	−	8.805	**0.000**					
BISAP	2.584	1.917	−	3.483	**0.000**	1.622	1.114	−	2.363	**0.012**
HGB	0.762	0.638	−	0.910	**0.003**					
Lymphocyte	0.446	0.251	−	0.792	**0.006**					
Creatinine	1.537	1.179	−	2.004	**0.002**					
Albumin	0.968	0.946	−	0.990	**0.005**					
Procalcitonin	1.029	0.988	−	1.070	0.167					

Note: Statistical significance is shown in bold-faced type (*p* < 0.05). Abbreviations: CONUT = Controlling Nutritional Status; BISAP = Bedside Index for Severity in Acute Pancreatitis; HGB = hemoglobin.

## Data Availability

While some or all datasets generated and/or analyzed during the current study are not publicly available, they can be made available from the Ethics Committee of the Haseki Training and Research Hospital upon reasonable request.
